# Authors’ Reply: The Anemia Risk Warning Model Based on a Noninvasive Method: Key Insights and Clarifications

**DOI:** 10.2196/74333

**Published:** 2025-04-22

**Authors:** Yahan Zhang, Yi Chun, Liping Tu, Jiatuo Xu

**Affiliations:** 1 Traditional Chinese Medicine College Shanghai University of Traditional Chinese Medicine Shanghai China

**Keywords:** anemia, hemoglobin, spectroscopy, machine learning, risk warning model, Shapley Additive Explanation

We sincerely appreciate your thoughtful and constructive comments on our recent paper exploring noninvasive anemia diagnosis through facial visible light reflectance spectroscopy and machine learning [1,2]. Your attention and insightful suggestions greatly guide our efforts to enhance this work. We address your key points below, offering clarifications as needed. You noted that uncontrolled factors like smoking and nutrition might influence anemia severity and spectral data. While our initial focus was validating this approach by controlling core variables (eg, age and gender), we plan to broaden future data collection to include these factors, analyze their effects, and integrate them into our model to improve reliability. You also emphasized how skin types, lighting, and individual variations could affect reflectance. We standardized conditions and preparation to minimize variability yet acknowledge our sample’s limited diversity. Given hemoglobin’s specific absorption peaks, we hypothesize that certain wavelength bands may be less skin tone–dependent. To explore this, we intend to collect data from diverse regions and skin types, aiming to identify such bands and enhance model applicability.

We value your recognition of our SVM model’s performance and your concern about overfitting due to sample size. Though it outperformed other algorithms, we are addressing this limitation by gathering more data via multicenter collaboration, applying 10-fold cross-validation, and validating with external datasets to boost accuracy and robustness. Our study focused on establishing facial spectral imaging’s potential for anemia detection, but comparative analysis could better assess its clinical utility. We are particularly intrigued by multimodal integration, such as combining spectroscopy with photoplethysmography (PPG) to improve diagnostic precision. Future work will involve comparative experiments to evaluate metrics like sensitivity and specificity, exploring whether synergy yields superior results. However, PPG indirectly reflects hemoglobin via light intensity changes, suiting routine monitoring, whereas spectroscopy directly measures hemoglobin concentration through reflectance spectra, fitting clinical diagnosis and research. Thus, stand-alone spectroscopy for noninvasive hemoglobin measurement holds significant promise, which we aim to further investigate.

We are encouraged by your recognition of this technique’s potential for community screening and personalized care. Thank you again for your rigorous review and profound insights. We look forward to further discussion and sharing progress or collaboration opportunities as our research advances.

## Abbreviations

PPG: photoplethysmography
